# Polymorphism in asparagine synthetase is associated with overall survival of hepatocellular carcinoma patients

**DOI:** 10.1186/s12876-017-0635-4

**Published:** 2017-06-19

**Authors:** Wei Li, Chengwei Dong

**Affiliations:** 1 Department of Interventional Radiology, the Affiliated Hospital of Qingdao University, Qingdao, Shandong 266003 China; 2 Department of Hepatobiliary Surgery, Weifang People’s Hospital, Weifang, Shandong 261041 China

**Keywords:** Hepatocellular carcinoma, ASNS, Realtime PCR, Association study, Survival analysis

## Abstract

**Background:**

Recently, it is reported that asparagine synthetase (ASNS) is an independent predictor of surgical survival in hepatocellular carcinoma (HCC) patients. It is also reported that activating transcription factor 6 (ATF6) expression is decreased in HCC patients. So in the present study, we explored the relationship between ASNS and ATF6, and whether ASNS expression was associated with HCC.

**Methods:**

ATF6 was over expressed in 3 HCC cell lines (HepG2, HepG2.2.15 and SMMC-7721). We then examined the mRNA levels of ASNS and ATF6 in 90 HCC patients, 77 chronic hepatitis B patients and 70 controls. We also genotyped 2 functional polymorphisms in *ASNS* in a case–control study.

**Results:**

The expression of ASNS was significantly elevated when ATF6 was over expressed. The expressions of these 2 genes were both decreased in HCC patients, and it was more significantly with ASNS. The mRNA levels of ASNS and ATF6 were positively correlated with each other. rs34050735 was associated with HCC in the case–control study (*P* = 0.003) and also an independent predictor of overall survival of HCC patients (*P* = 0.001).

**Conclusions:**

Taken together, these findings indicated that rs34050735 in *ASNS* may associate with HCC and may be a promising biomarker of HCC.

## Background

Hepatocellular carcinoma (HCC) is common cancer mortality worldwide. There are about 564,000 new cases of HCC each year throughout the world [1]. Recently, Zhang et al. reported that asparagine synthetase (ASNS) was an independent predictor of surgical survival and a potential therapeutic target in HCC [2]. Meanwhile, Wu et al. reported that HBsAg-negative healthy individuals and chronic hepatitis B (CHB) patients had higher ATF6 mRNA levels than HCC patients [3].

The *ASNS* gene encodes a protein involved in the synthesis of asparagine [4]. Asparagine is an essential amino acid for cell growth and survival. The transcription of *ASNS* is regulated by the nutritional status of the cell [5]. ASNS has been considered as a predictive biomarker in ovarian cancer [6], pancreatic cancer [7] and prostate cancer [8]. The *ATF6* gene encodes a transcription factor, acting as an unfolded protein response (UPR) transcriptional activator which regulates gene expression of endoplasmic reticulum (ER) chaperones, ER-associated proteins, and apoptotic genes [9, 10]. ATF6 works to alleviate ER stress by decreasing the amount of misfolded/unfolded proteins in the ER, or if this cannot be achieved, by initiating cell apoptosis [11]. As suggested by computational analysis (AliBaba 2 software), there is an ATF6 binding site in the promoter region of *ASNA* gene. So we speculated that ATF6 may regulate the expression of ASNS, and ASNA may also associated with HCC tumorigenesis. We carried out the present study to test this hypothesis.

## Methods

### Subjects, ethics, consent and permissions

The subjects enrolled in this study were constituted of 2 independent groups of patients.

The first was constituted of 90 HCC patients, 77 CHB patients and 70 non-HBV controls which were enrolled from the Weifang People’s Hospital from Jan 2011 to Nov 2014. The second was constituted of 337 HCC patients and 310 CHB patients enrolled from the same hospital from May 2005 to Jan 2010. Among the 337 HCC patients, clinical outcomes of 146 patients that had undergone surgical resection of a HCC tumor were recorded until October 2015, with a median follow-up time of 39.5 months (range 5.0–76.5 months).

HCC patients, CHB patients and non-HBV controls were defined as previously reported [3]. The main features of the subjects were summarized in Table 1. The study was carried out in accordance with the guidelines of the Helsinki Declaration after obtaining written informed consent from all the subjects and was approved by the ethics committee of the Weifang People’s Hospital. All patients consented to participate this study.

**Table 1:** Clinical features of the subjects included in the study

A. Realtime PCR Study			
	Non-HBV controls *n* = 70	CHB patients *n* = 77	HCC patients *n* = 90
Age, *y* mean ± SD	48.2 ± 7.5	49.1 ± 10.1	50.3 ± 9.5
Gender, n. (%)			
Male	39(55.7)	43(55.8)	50(55.6)
Famale	31(44.3)	34(44.2)	40(44.4)
Smoking, n. (%)			
Yes	30(42.9)	45(58.4)	55(61.1)
No	40(57.1)	32(41.6)	35(38.9)
Drinking, n. (%)			
Yes	33(47.1)	50(64.9)	73(81.1)
No	37(52.9)	27(35.1)	17(18.9)
B. Case–control Study			
	HCC	CHB	P
Number	337	310	
Age, *y* mean ± SD	44.7 ± 11.0	44.3 ± 12.3	0.67
Gender (male/female)	298/39	254/56	0.02
Smoking (Yes/No)	141/192	128/182	0.79
Drinking (Yes/No)	98/239	92/218	0.87
Family history of HCC (Yes/No)	58/279	14/296	<0.001

*HCC* hepatocellular carcinoma, *CHB* chronic hepatitis B

### Gene expression experiment

HepG2, HepG2. 2.15, and SMMC-7721 cells were were kindly gifts from professor Xiaopan Wu (National Laboratory of Medical Molecular Biology, Institute of Basic Medical Sciences, Chinese Academy of Medical Sciences, Beijing, China). The cells were propagated in MEM/NEAA or RPMI-1640 medium with 10% fetal calf serum. All cells were maintained with 5% CO2 at 37 °C. We seeded 2 × 10^5^ HepG2, HepG2. 2.15, or SMMC-7721 cells each well in 24-well plates. The ATF6 expression plasmid was constructed as previous reported [3]. Half Wells were transfected with ATF6 expression plasmid by Lipofectamine™ 2000 (Invitrogen, Carlsbad, CA). The rest half were non-transfected cells regarded as controls. All transfections were repeated 3 times. The primers used for qPCR and detailed qPCR methods were according to previously reported [2, 3]. Total RNA was extracted from the peripheral blood of 90 HCC patients, 77 CHB patients and 70 non-HBV controls and mRNA levels of ATF6 and ASNS were tested. The detailed qPCR methods were the same as above mentioned.

### Plasmids and luciferase assay

We constructed a pGL3-Basic (Promega, Madison, WI) reporter plasmid encompassing −395 to +145 bp of ASNS promoter. The ATF6 eukaryotic expression plasmid and FLAG control plasmid were gifts from Dr. Wu (Institute of Basic Medical Sciences, Chinese Academy of Medical Sciences). The luciferase assay was performed as previously reported [3] in HepG2 cells.

### SNP selection and genotyping

Genomic DNA were extracted from peripheral blood using the salting-out protocol. Using the NCBI dbSNP database (http://www.ncbi.nlm.nih.gov/snp/), potential functional SNPs (SNPs in promoter region and mRNA sequence) with minor allele frequency (MAF) greater than 0.05 for the Han Chinese Beijing population were selected. Only 2 SNPs were found, namely rs1049674 (nonsynonymous coding) and rs34050735 (5’UTR). These 2 SNPs were genotyped using TaqMan method (Applied Biosystems, Foster City, CA), according to the manufacture’s protocols. All the samples were successfully genotyped.

### Statistical analysis

ANOVA was used to examine the differences in mRNA expression levels between different groups. By using the χ2 test, we tested whether the genotype distributions of SNP were in the Hardy–Weinberg equilibrium (HWE). We used 2 × 2 or 2 × 3 contingency tables for comparing allele and genotype frequencies between different groups. We calculated the linkage disequilibrium values (r2, D’) and the haplotype estimation using the SHEsis online software [12]. The associations between overall survival and demographic characteristics, and rs1049674 and rs34050735 were estimated using the Kaplan–Meier method. A survival curve was drawn with the Kaplan–Meier method for each genotype. *P* < 10.05 was the criterion for statistical significance. All statistical analyses were performed using the Statistical Package for the Social Sciences (SPSS), version 15.0 (SPSS Inc., Chicago, Illinois).

## **Results**

### ASNS was positively regulated by ATF6

**Fig. 1:**
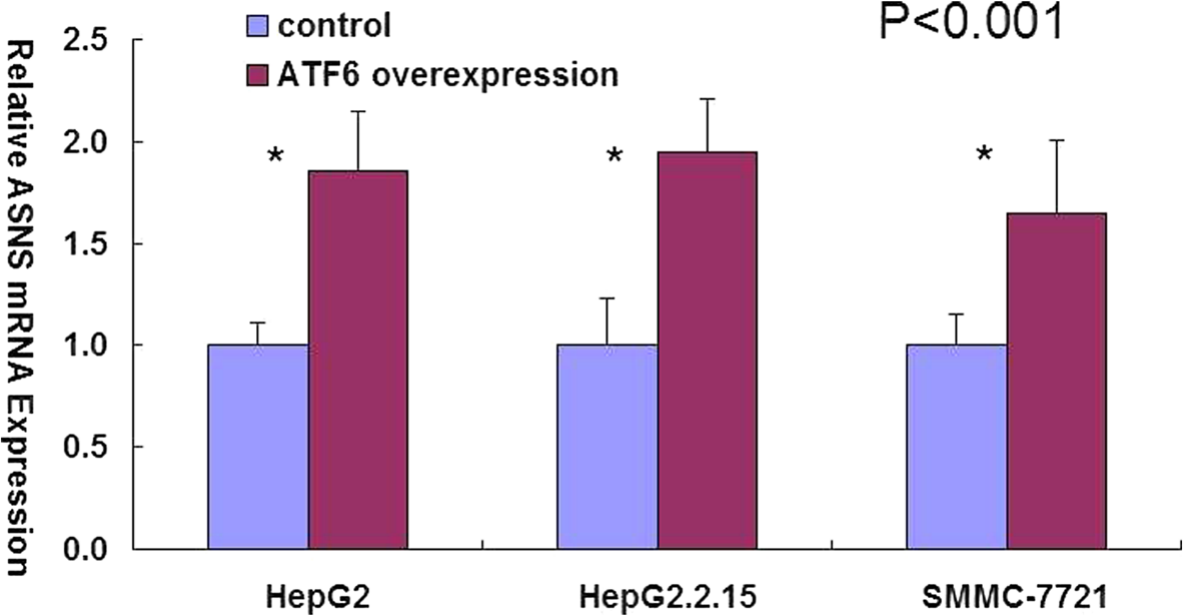
Relative ASNS mRNA Expression in HepG2, HepG2. 2.15, and SMMC-7721 cells. Final abundance figures were adjusted to yield an arbitrary value of 1 for non-transfected cells. Data are means ± SD. *P < 0.001

We transiently transfected HepG2, HepG2.2.15, and SMMC-7721 cells with ATF6 expression plasmid, and then examined the mRNA expression of ASNS. Final abundance figures were adjusted to yield an arbitrary value of 1 for non-transfected cells. The result showed that when ATF6 was over expressed, the mRNA level of ASNS was elevated by 1.86-fold, 1.95-fold and 1.65-fold in HepG2, HepG2.2.15, and SMMC-7721 cells, respectively (*P* < 0.001) (Fig. 1).

**Fig. 2:**
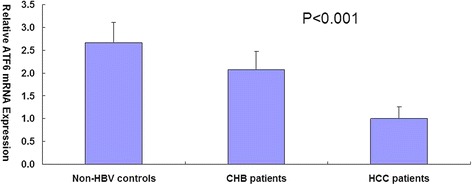
Quantification of ATF6 mRNA expression by real-time PCR. GAPDH was used as an internal control gene. Final abundance figures were adjusted to yield an arbitrary value of 1 for HCC patients. Data are means ± SD

**Fig. 3:**
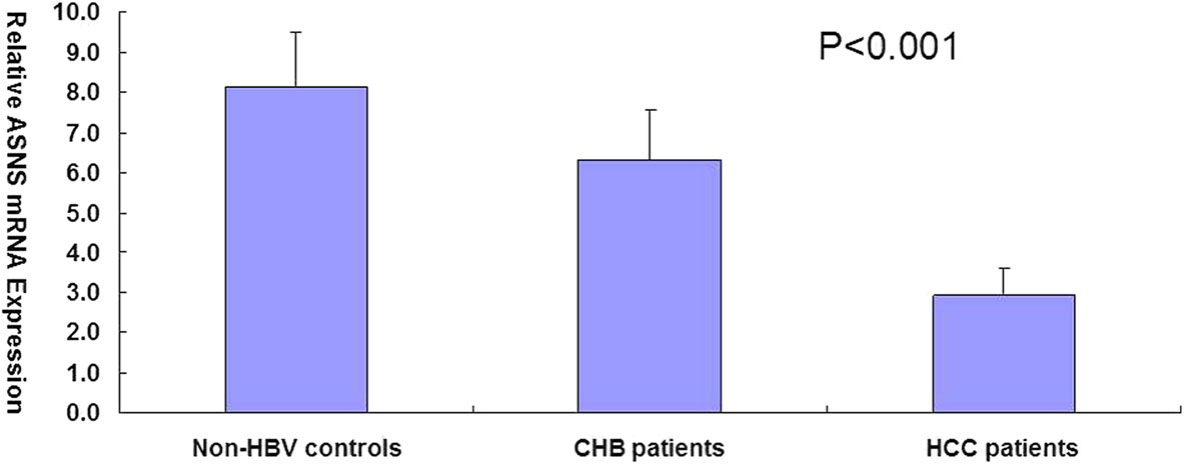
Quantification of ASNS mRNA expression by real-time PCR. GAPDH was used as an internal control gene. Data are means ± SD

We then examined levels of total ATF6 and ASNS mRNA using quantitative realtime PCR. The mRNA levels of ATF6 and ASNS were measured. As shown in Table 1, the 3 groups of subjects had similar age and sex distribution, but CHB and HCC groups had higher smoking and drinking ratio than non-HBV controls. Final abundance figures were adjusted to yield an arbitrary value of 1 for ATF6 expression level in HCC patients (Figs. 2 and 3). The result showed that non-HBV controls and CHB patients had 2.67-fold and 2.08-fold higher ATF6 mRNA levels than HCC patients (*P* = 5.38E-79), and 2.78-fold and 2.16-fold higher ASNS mRNA levels than HCC patients (*P* = 9.05E-82). We also found that ASNS expression level was positively correlated with ATF6 level (r2 = 0.98).

To further confirm whether ATF6 regulated *ASNS* promoter, we transiently transfected HepG2 cells with pGL3-ASNS promoter together with ATF6 expressing plasmid or FLAG control plasmid. The result showed that the ATF6 expressing plasmid group had 1.59-fold higher luciferase activity compared with FLAG control group (*P* = 0.002). This result indicated that ATF6 could indeed positively regulate the promoter region of *ASNS* gene.

### Case–control study of SNPs in *ASNS* gene

We next conducted genotyping experiments for the 2 ASNS polymorphisms in the case–control samples. Genotype distributions of the studied SNPs were in HWE in both cases and controls. The genotype distributions and allelic frequencies of ASNS polymorphisms in CHB and HCC patients were represented in Table 2. The frequency of T allele of rs34050735 was 16.5% in HCC patients vs. 10.8% in CHB patients (*P* = 0.003, OR = 1.63, 95%CI = 1.18–2.25). The Cochran-Armitage trend test (assuming an additive model for T allele) revealed an allele dose-dependent association of rs34050735 with HCC (*P* = 0.005), with decreased OR of 0.71 and 0.44 for GT and GG genotypes, respectively.

**Table 2:** Genotype distributions of 2 SNPs in ASNS gene

	Allele, n (ratio)				Genotype, n (ratio)			Cochran Armitage trend test	logistic regression^*#*^
	1/2	1	2	P/OR (95% CI)	11	12	22	P	P/OR (95% CI)
rs1049674	T/A								
HCC (*n* = 337)		57(0.085)	617(0.915)	0.72	2(0.006)	53(0.157)	282(0.837)	0.72	0.55
CHB (*n* = 310)		49(0.079)	571(0.921)	1.08(0.72–1.60)	3(0.010)	43(0.139)	264(0.852)		1.14(0.75–1.72)
rs34050735	T/G								
HCC (*n* = 337)		111(0.165)	563(0.835)	0.003	13(0.039)	85(0.252)	239(0.709)	0.005	0.005
CHB (*n* = 310)		67(0.108)	553(0.892)	1.63(1.18–2.25)	6(0.019)	55(0.177)	249(0.803)		1.59(1.15–2.20)

^*#*^ *P* values were adjusted for age, gender, smoking drinking and family history of HCC by binary logistic regression under additive model

We then used binary logistic regression to adjust for confounding factors as age, gender, smoking drinking and family history under additive model, and the results showed that rs34050735 was still independently associated with HCC (*P* = 0.005, OR = 1.59, 95%CI = 1.15–2.20). While the other SNP rs1049674 was not associated with HCC under any model. We analyzed the degree of LD for these 2 SNPs, and found there was no apparent LD (D’ ≤ 0.05, r^2^ ≤ 0.002). Table 3 shows 4 haplotypes constructed by these 2 SNPs. The A-T haplotype was associated with HCC (*P* = 0.02).

**Table 3:** Common haplotypes constructed with SNPs rs1049674 and rs34050735 in ASNS gene

Haplotypes	HCC (*n* = 337)	CHB (*n* = 310)	P	OR (95%CI)
A-G	520.76(0.773)	508.74(0.821)	0.09	0.79(0.60–1.04)
A-T	96.24(0.143)	62.26(0.100)	0.02	1.52(1.08–2.13)
T-G	42.24(0.063)	44.26(0.071)	0.58	0.88(0.57–1.37)
T-T^a^	14.76(0.022)	4.74(0.008)		

^a^Haplotypes with frequency < 0.03 was ignored in analysis

### Survival analysis

**Fig. 4:**
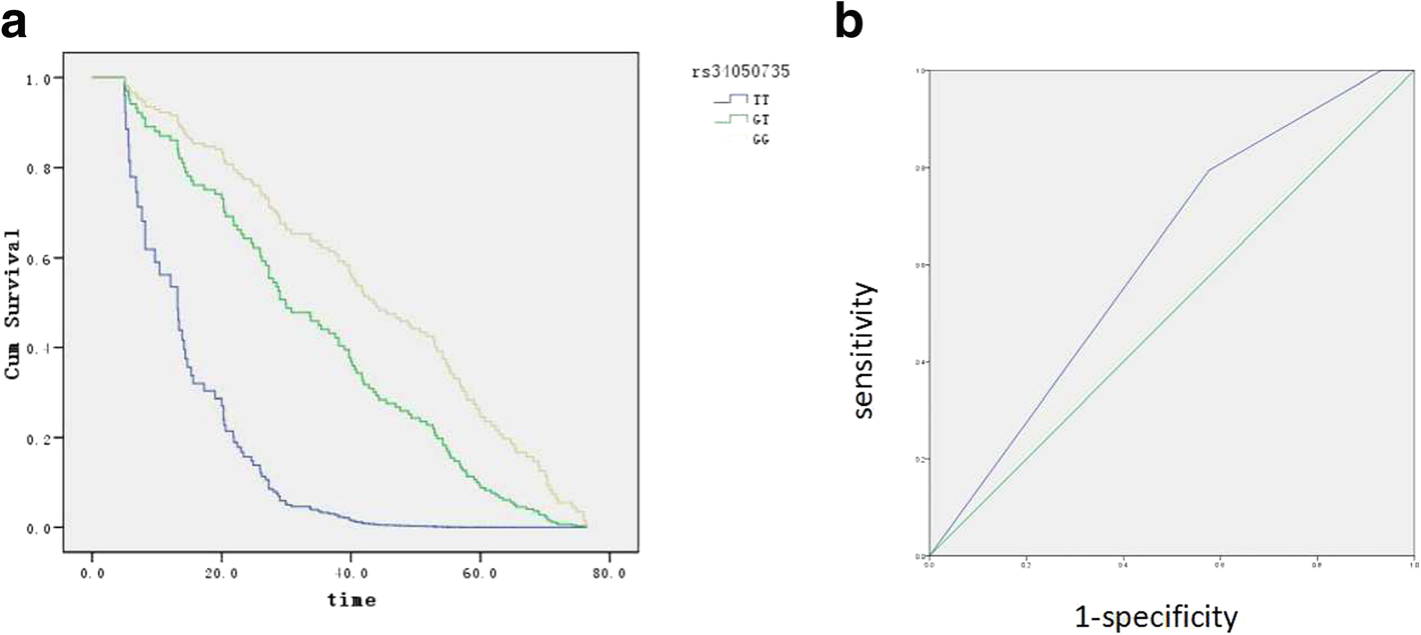
rs34050735 genotype and HCC survival. **a** Kaplan–Meier survival curves of overall survival for HCC patients by rs34050735 genotype. **b** Using ROC curve to establish the prognosis of HCC patients

Finally, we asked the question whether these 2 SNPs influencing overall survival of HCC patients. Among the 146 HCC patients with clinical outcomes, 127 patients were died and were included in the final analysis. As shown in Table 4 and Fig. 4, SNP rs34050735 was significantly associated with overall survival (*P* = 0.001). Patients who carried the TT genotype had a significantly shorter survival time compared to those with the GT or GG genotypes. We used Receiver Operating Characteristic (ROC) curve to establish the prognosis of HCC patients. According to the ROC curve, patients whose survival time more than or equal to 38 months were defined as the better group, survival time less than 38 months were defined as the poor group (*P* = 0.025).

**Table 4:** Clinical characteristics and their prediction of overall survival in 127 HCC patients

Characteristics	Number	Survival Time, *m* mean (95%CI)	P	OR (95%CI)
Gender			0.68	1.12(0.65–1.93)
male	107	39.1(35.1–43.2)		
female	20	41.8(31.0–52.6)		
Smoking			0.24	0.72(0.41–1.25)
No	86	40.8(36.1–45.4)		
Yes	41	37.0(30.7–43.4)		
Drinking			0.90	1.04(0.57–1.89)
No	97	40.3(36.0–44.6)		
Yes	30	37.1(29.3–45.0)		
Family history of HCC			0.16	0.65(0.36–1.19)
No	114	40.6(36.7–44.6)		
Yes	13	30.2(18.0–42.4)		
rs1049674			0.829	0.80(0.10–6.17)
TT	1	54.2		
TA	22	33.6(24.0–43.2)		
AA	104	40.7(36.6–44.8)		
rs34050735			0.001	7.21(2.30–22.6)
TT	4	15.2(−2.4–32.7)		
TG	35	34.4(27.7–41.1)		
GG	88	42.7(38.2–47.2)		
Vascular invasion			0.51	0.86(0.55–1.34)
No	78	39.2(34.4–44.1)		
Yes	49	40.1(34.1–46.0)		
Differentiation			0.28	1.27(0.82–1.97)
I + II	65	38.9(33.4–44.4)		
III + IV	62	40.3(35.1–45.4)		
TNM stage			0.53	0.88(0.60–1.30)
I + II	75	39.6(34.8–44.5)		
III + IV	52	39.4(33.4–45.5)

## **Discussion**

As an UPR-stimulating gene, ATF6 plays an important role in tumor genesis, and *ASNS* gene is important in tumor genesis due to its function of synthesis of asparagine, which is an essential amino acid for normal tissue or tumor growth. We found in this study that the mRNA levels of ASNS and ATF6 were positively correlated with each other, and the decrease of ASNS mRNA level in HCC patients was greater than ATF6. So as the disease progressed, the ASNA mRNA level was negatively correlated with the severity of HCC. This result may have potential application value as ASNA might be a useful diagnoses bio-marker of HCC.

Recent progresses have been made in determining the role of ATF6 and ASNS in varies tumors. ATF6 are reported to contribute to enhanced viability in glioblastoma [13], and important for survival of melanoma cells undergoing ER stress [14]. Knockdown of ASNS suppresses cell growth in human melanoma cells and epidermoid carcinoma cells [15]. Polymorphisms of asparaginase pathway genes are related with asparaginase-related complications in children with acute lymphoblastic leukemia [16], probably by affecting early response to treatment [17]. Down-regulation of ASNS induces cell cycle arrest and inhibits cell proliferation of breast cancer [18].

In the present study, we replicated Wu’s work that ATF6 mRNA level decreased in turn from non-HBV controls to CHB patients and HCC patients [3]. However, Zhang’s work revealed that the expression of ASNS was higher in HCC tumor tissues [2]. Our results showed that ASNS mRNA decreased in the peripheral blood of HCC patients, which was deviated to Zhang’s. On the other hand, although Zhang’s work showed ASNS was higher expressed in HCC tumor tissue, ASNS seemed to have antitumor effect, for patients with low ASNS expression levels had a poor prognosis and ASNS significantly inhibited the proliferation, migration and tumourigenicity of HCC cells. We speculated that the essential role of ASNS (synthesis of asparagine) made it a double-edge sword to HCC, whether it exercise good or bad effect on tumourigenicity depended on the complicated interaction between ASNS and other related genes. So further studies are needed to clarify the exact role of ASNS in tumourigenicity.

rs34050735 is in the 5’UTR region of ASNS gene. The 5’UTR region maybe the target of transcription factor. So further studies are needed to clarify the exact functional role of rs34050735.

Several limitations of the present study need to be addressed. Further tests of ATF6 and ASNS mRNA levels in HCC tumor tissues and corresponding non tumor tissues should be done. The protein levels of ATF6 and ASNS should also be tested.

## Conclusions

We found in the present study that ASNS and SNP in this gene may associate with HCC and be a promising bio-marker of HCC.

## Abbreviations

ASNS: Asparagine synthetase
CHB: Chronic hepatitis B
ER: Endoplasmic reticulum
HCC: Hepatocellular carcinoma
MAF: Minor allele frequency
UPR: Unfolded protein response
